# Frequency and Factors of Indeterminate QuantiFERON-TB Gold In-Tube and QuantiFERON-TB Gold PLUS Test Results in Rheumatic Diseases

**DOI:** 10.3390/jcm10194357

**Published:** 2021-09-24

**Authors:** Sung Soo Ahn, Hyung Woo Kim, Younhee Park

**Affiliations:** 1 Department of Internal Medicine, Yongin Severance Hospital, Yonsei University College of Medicine, Yongin 16995, Korea; saneth@yuhs.ac; 2 Department of Internal Medicine, College of Medicine, Institute of Kidney Disease Research, Yonsei University, Seoul 03722, Korea; drhwint@yuhs.ac; 3 Department of Laboratory Medicine, Severance Hospital, Yonsei University College of Medicine, Seoul 03722, Korea

**Keywords:** QuantiFERON-TB Gold In-Tube, QuantiFERON-TB Gold PLUS, rheumatic diseases, indeterminate, factors

## Abstract

We compared the results and differences of indeterminate rates between the QuantiFERON-TB Gold In-Tube (QFT-GIT) and QuantiFERON-TB Gold PLUS (QFT-PLUS) tests in patients with rheumatic diseases and analyzed the associated factors. Data of patients with rheumatic diseases who had undergone the QFT-GIT or QFT-PLUS test were used, and information regarding patient demographics, primary diagnosis, laboratory results, and medications was collected. Furthermore, indeterminate result rates of the patient cohort and healthy controls were also compared. A total of 177 (43.4%) and 231 (56.6%) patients had undergone QFT-GIT and QFT-PLUS tests, respectively. Among them, four (2.3%) and seven (3.0%) patients had indeterminate results, which did not differ between the QFT-GIT and QFT-PLUS groups. Indeterminate results were significantly higher among patients with rheumatic diseases than in healthy controls (2.7% vs. 0.2%, *p* < 0.001). Multivariate logistic regression revealed that the lymphocyte count (hazard ratio (HR) 0.998, 95% confidence interval (CI) 0.997, 1.000; *p* = 0.012) and albumin level (HR 0.366, 95% CI 0.150, 0.890; *p* = 0.027) were predictive of indeterminate results. A lymphocyte count of ≤810/mm^3^ and an albumin level of ≤3.7 mg/dL were capable of discriminating between indeterminate and determinate results. The QFT-GIT and QFT-PLUS tests have comparable diagnostic performances in patients with rheumatic diseases. Decreased lymphocyte and albumin levels contribute to indeterminate results.

## 1. Introduction

Tuberculosis (TB) is a highly contagious disease caused by *Mycobacterium tuberculosis* (MTB) infection and is a substantial public health burden. Given that more than 10 million people are affected with TB annually, it stands as one of the leading causes of death worldwide [1]. Latent tuberculosis infection (LTBI) is defined as being infected with MTB in the absence of active signs and symptoms of TB. This is considered a major risk factor for developing active TB [2]; therefore, appropriate screening of LTBI in high-risk individuals is considered crucial in preventing the community transmission of TB. An interferon-gamma release assay (IGRA) is a laboratory test used to diagnose LTBI by measuring the release of inflammatory cytokines, such as interferon-γ (IFN-γ), after mycobacterial antigen exposure [3]. After the first approval of TSPOT.TB as a commercially available IGRA in 2004, QuantiFERON-TB (QFT) Gold was adopted for clinical use in 2005 and is now being widely used in medical practice for LTBI screening. In particular, advanced new generation QFT tests—QuantiFERON-TB Gold In-Tube (QFT-GIT) and QuantiFERON-TB Gold PLUS (QFT-PLUS) tests—have been developed and used in clinical settings [4]. The QFT-PLUS test, which was approved by the FDA in 2017, is the latest QFT assay. The main difference between the QFT-PLUS and QFT-GIT tests is that two TB antigen tubes, TB1 and TB2, are utilized to measure the CD4+ T cell and CD8+ T cell immune responses, respectively, in the former. The individual evaluation of CD4+ and CD8+ T cell IFN-γ production is thought to help in assessing adaptive immunity more precisely than that with a combined evaluation [5]. Notably, the literature has suggested that the QFT-PLUS test correlates well with the previous version of the QFT test while having an improved diagnostic performance, particularly in terms of sensitivity and specificity. Additionally, it is preferred for immunocompromised patients such as those with human immunodeficiency virus (HIV) infection [6,7,8]. 

The underlying pathogenesis of rheumatic diseases is characterized by the development of aberrant immunity [9]. Consequently, the unbalanced pro- and anti-inflammatory immune response is a typical feature of rheumatic diseases, and therapeutic agents generally target excessive inflammation. Previous studies have demonstrated that indeterminate IGRA results can be found in the presence of altered immunity or the use of immunosuppressive drugs [10,11,12]. Thus, it was suggested that indeterminate results are more frequently reported in patients with rheumatic diseases. A single-center study that evaluated the prevalence of indeterminate rates using QFT-GIT tests showed that the overall frequency of an indeterminate IGRA was 6.8% [13]. Conversely, an analysis of patients tested using QFT-GIT tests in a large United States health system reported that indeterminate results were found in 5.3% of patients with chronic inflammatory disease, and these patients were 2.4 times more likely to have indeterminate results than hospital employees [14]. 

Given that QFT-PLUS tests, which comprise four tubes to measure IFN-γ, measure helper and cytotoxic T cell responses separately and have a distinct standard to define indeterminate results compared to those QFT-GIT tests, it is possible that the diagnostic performance of the two assays could differ in patients with rheumatic diseases. However, to the best of our knowledge, no study on this has been conducted. Additionally, the influence of clinical and laboratory parameters associated with indeterminate results is still inconclusive in this population. Therefore, this study aimed to analyze whether there is a difference in indeterminate rates among patients with rheumatic diseases tested with QFT-GIT and QFT-PLUS tests and the factors affecting these results.

## 2. Materials and Methods

### 2.1. Data Collection 

**Figure 1:**
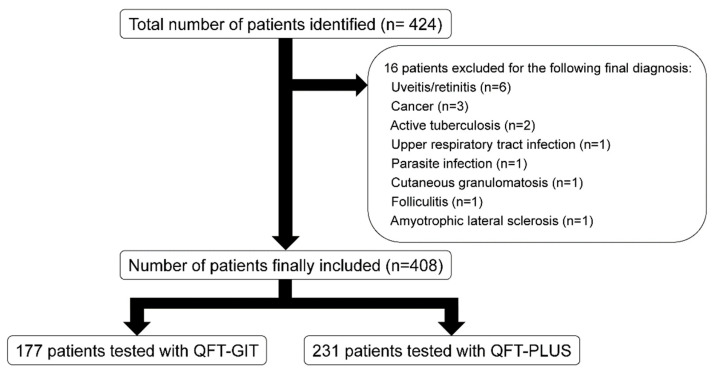
Patient selection and exclusion. QFT-GIT: QuantiFERON-TB Gold In-Tube; QFT-PLUS: QuantiFERON-TB Gold PLUS.

This retrospective analysis was performed using data of patients who underwent QFT-GIT and QFT-PLUS tests at Severance Hospital during the period of April 2018 to March 2019. In Severance Hospital, the QFT-GIT test was utilized to diagnose LTBI until September 2018, and the QFT-PLUS test was introduced after October 2018. During this period, 4253 patients with IGRA data were identified from the hospital’s Clinical Data Repository System. These included 424 patients with a primary diagnosis of rheumatoid arthritis (RA), ankylosing spondylitis, Behçet disease, systemic lupus erythematosus (SLE), adult-onset Still’s disease, anti-neutrophil cytoplasmic antibody-associated vasculitis (either microscopic polyangiitis, granulomatosis with polyangiitis, or eosinophilic granulomatosis with polyangiitis), polyarteritis nodosa, psoriatic arthritis, dermatomyositis (DM) or polymyositis, polymyalgia rheumatica, systemic sclerosis, and immunoglobulin G4-related disease. After reviewing medical records, it was found that 16 patients had a diagnosis unrelated to or incomplete for diagnosing the rheumatic diseases investigated. Finally, a total of 408 patients were included in the study (Figure 1). 

This study was approved by the Severance Hospital Institutional Review Board (4-2021-0353), and all relevant procedures were performed in accordance with the Declaration of Helsinki. The requirement to obtain informed consent from the patients was waived as this was a retrospective study.

### 2.2. Analysis of Patient Data and Medication 

Patient data consisted of age, sex, primary diagnosis, laboratory data at the date of QFT-GIT or QFT-PLUS testing, and the results of the IGRA. Laboratory data consisted of white blood cell, neutrophil, lymphocyte, and platelet counts, as well as hemoglobin, blood urea nitrogen, creatinine, albumin, aspartate aminotransferase, alanine aminotransferase (ALT), and C-reactive protein (CRP) levels and erythrocyte sedimentation rate (ESR). Information on medications—glucocorticoid, methotrexate, hydroxychloroquine, sulfasalazine, tacrolimus, leflunomide, 5-acetylsalicylic acid, azathioprine, mycophenolate mofetil, cyclophosphamide, cyclosporine, biologic and targeted synthetic disease-modifying antirheumatic drugs—that the patients were being administered for disease control was also collected by a retrospective chart review.

### 2.3. QFT-GIT and QFT-PLUS Assay and Healthy Controls

Both the QFT-GIT and QFT-PLUS (Qiagen, Hilden, Germany) tests were performed according to the instructions provided by the manufacturer using a standardized protocol in the Department of Laboratory Medicine, Division of Diagnostic Immunology. Interpretation of IGRA results was conducted as described previously [15]. The South Korean government mandates healthcare-related professionals working in hospitals to undergo testing using an IGRA to evaluate the presence of LTBI before employment. The results of the IGRA from individuals who were subject to the employment check-up were utilized to evaluate the difference in indeterminate rates between patients with rheumatic diseases and healthy controls. Data one year prior to the introduction of the QFT-PLUS test (by QFT-GIT, *n* = 2511) and data one year after the adoption of the QFT-PLUS test (*n* = 1614) were analyzed.

### 2.4. Statistical Analysis

All statistical analyses were conducted using MedCalc version 19.6.4 (MedCalc Software, Ostend, Belgium). Continuous variables are represented as mean ± standard deviation, whereas categorical variables are shown as frequencies and percentages. Statistical differences between continuous variables were evaluated by Student’s t-test, while comparison of categorical variables was conducted utilizing a chi-square test or Fisher’s exact test for two groups, or a chi-square test for trends in more than three groups. Multivariate logistic regression analysis using a forward entry method including significant variables in the univariate analysis was conducted to evaluate factors associated with indeterminate results. The ideal cut-off values for predicting indeterminate results were elucidated by receiver operating characteristic (ROC) curve analysis, and calculation of the relative risk (RR) was performed using contingency tables and a chi-square test. A two-tailed *p*-value of <0.05 was considered statistically significant.

## 3. Results

### 3.1. Comparison of Patient Characteristics and Medications between the QFT-GIT and QFT-PLUS Groups

Among the 408 patients with rheumatic diseases, 177 (43.4%) and 231 (56.6%) patients had undergone QFT-GIT and QFT-PLUS tests, respectively. No differences were observed with respect to age and the distribution of primary diagnoses. The frequency of males was higher in the QFT-PLUS group than in the QFT-GIT group (39.4% vs. 29.4%, *p* = 0.036). Concerning laboratory data, although the ESR was found to be elevated in the QFT-GIT group, other parameters were comparable between the two groups. The results of the IGRA were similar between the QFT-GIT and QFT-PLUS groups; indeterminate results were found in 2.3% (4/177 patients) and 3.0% (7/231 patients) of patients in the QFT-GIT and QFT-PLUS groups, respectively (Table 1). 

When the use of medication was compared between the groups, patients in the QFT-GIT group were more often prescribed glucocorticoids and tacrolimus than patients in the QFT-PLUS group. However, the use of other medications did not differ significantly (Table 2). 

### 3.2. Indeterminate IGRA Results in Patients with Rheumatic Diseases and Healthy Controls

On comparing the rates of indeterminate results between patients with rheumatic diseases and healthy controls tested using QFT-GIT or QFT-PLUS during the employment check-up, it was found that the rates were significantly higher in patients with rheumatic diseases than in controls (*p* < 0.001, Table 3). 

### 3.3. Patients with an Indeterminate IGRA and Factors Associated with the Occurrence of Indeterminate Results

In total, eleven patients (2.7%) had indeterminate results. Of these, eight patients (72.7%) were female. The primary diagnoses were SLE for four patients, RA for three patients, and DM for three patients. There was a large variability in the laboratory data: lymphocyte count, 30–1830 mm^3^; albumin level, 2.6–4.5 mg/dL; ESR, 17–120 mm/h; and CRP level, 0.3–341.1 mg/L, and only two patients were treatment-naïve. Additionally, the analysis of IFN-γ in separate tubes indicated that two of the patients had high IFN-γ levels detected in the Nil tube, whereas the remaining nine cases had decreased IFN-γ levels in the Mitogen tube (Table 4). 

In the univariate logistic regression analysis, it was determined that lymphocyte and platelet counts, hemoglobin, albumin, ALT, and CRP levels, and ESR were associated with an indeterminate IGRA result. The inclusion of these variables in the multivariate logistic regression analysis revealed that only the lymphocyte count (hazard ratio (HR) 0.998, 95% confidence interval (CI) 0.997, 1.000; *p* = 0.012) and albumin level (HR 0.366, 95% CI 0.150, 0.890; *p* = 0.027) were independent predictive factors (Table 5).

### 3.4. Estimating Ideal Cut-Off Value of Lymphocyte Count and Albumin Levels for Discrimination of Indeterminate Results

**Figure 2:**
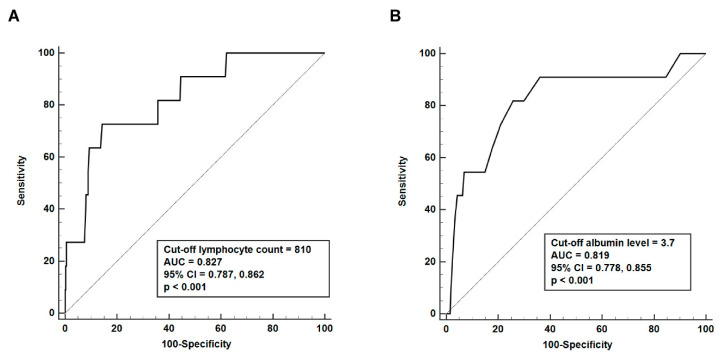
Cut-off values of absolute lymphocyte count and albumin levels associated with indeterminate results. In the receiver operating characteristic curve analysis, (**A**) a lymphocyte count of ≤810 mm^3^ or (**B**) an albumin level of ≤3.7 mg/dL was capable of discriminating between indeterminate and determinate results of interferon-gamma release assays. AUC: area under the curve; CI: confidence interval.

In the ROC curve analysis, the area under the ROC curve for a lymphocyte count of ≤810/mm^3^ was 0.827 (95% CI 0.787, 0.862; *p* < 0.001), and for an albumin level of ≤3.7 mg/dL, it was 0.819 (95% CI 0.778, 0.855; *p* < 0.001), for discriminating between indeterminate and determinate results (Figure 2). 

**Figure 3:**
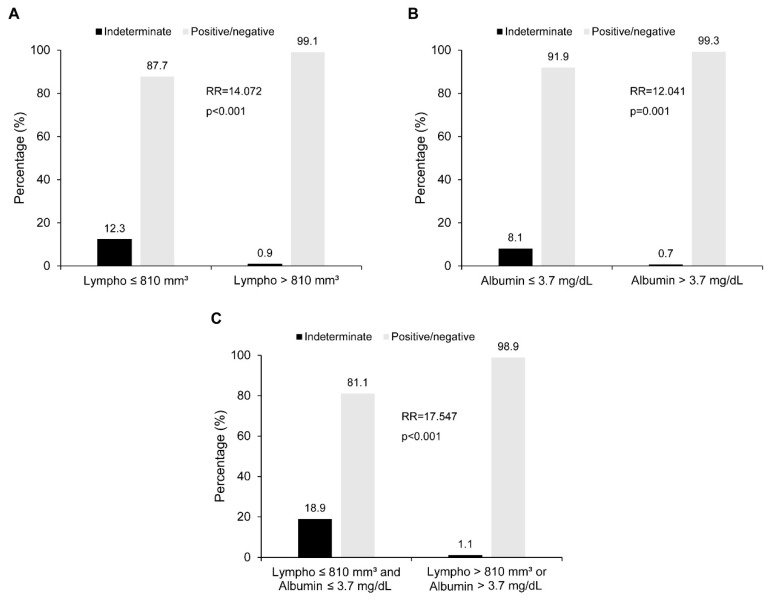
Relative risk of indeterminate results according to lymphocyte count and albumin level. Patients with (**A**) a lymphocyte count of ≤810 mm^3^ and (**B**) an albumin level of ≤3.7 mg/dL had a significantly higher risk of indeterminate results being reported than those who did not meet these criteria. In addition, the RR increased in those with both (**C**) a lymphocyte count of ≤810 mm^3^ and an albumin level of ≤3.7 mg/dL. RR: relative risk.

Furthermore, the RR of having indeterminate results in those with a lymphocyte count of ≤810/mm^3^ and an albumin level of ≤3.7 mg/dL was found to be 14.072 (95% CI 3.834, 51.644; *p* < 0.001) and 12.041 (95% CI 2.642, 54.866; *p* = 0.001), respectively. The combination of a lymphocyte count of ≤810/mm^3^ and an albumin level of ≤3.7 mg/dL increased this RR to 17.547 (95% CI 5.386, 57.169; *p* < 0.001) (Figure 3).

## 4. Discussion

An indeterminate IGRA result is commonly found in patients with rheumatic diseases, with an immunocompromised state, or in a critically ill condition [14,16,17]. On the other hand, indeterminate results cause significant uncertainty when making a clinical decision on whether treatment for LTBI is required. On analyzing the data of 408 patients with rheumatic diseases tested with QFT-QIT or QFT-PLUS, it was observed that the fourth generation of the QFT test (the QFT-PLUS) had similar outcomes regarding indeterminate results to those of its predecessor, the QFT-GIT test, implying that the diagnostic utility of both assays is comparable. Additionally, in line with previous studies, we demonstrated that indeterminate results are more frequently found in patients with rheumatic diseases than in healthy controls. Importantly, it was also revealed that lymphocyte counts and albumin levels are independent factors that could influence the indeterminate results of the QFT-GIT and QFT-PLUS tests.

Despite the decreasing burden of TB in South Korea, it is still a major concern owing to the higher rates of incidence and prevalence compared to those in Western countries. Furthermore, LTBI was reportedly found in over 30% of the general population according to a recent national TB statistics report [18]. In the present study, it was found that 20.6% of patients (84 out of 408 patients) had positive IGRA results. The relatively low positive rates of IGRAs compared to those in the general population might be attributed to the fact that LTBI is more prevalent in males and elderly populations [19]; meanwhile, rheumatic diseases are more frequent in females and uncommon in elderly individuals. Moreover, we found that the overall indeterminate result rate was 2.7% in patients tested using QFT-GIT and QFT-PLUS. However, this rate seems to be somewhat lower compared to those reported previously. Surprisingly, among the healthcare providers (4125 individuals) who underwent testing using IGRAs (QFT-GIT or QFT-PLUS) in our hospital for an employment check-up, only nine had indeterminate results. This extremely low rate of indeterminate results among healthy controls in our study seems to be similar to that of a recent publication that reported an indeterminate rate of 0.1% with QFT-GIT tests in healthcare workers, emphasizing that an indeterminate IGRA result in immunocompetent individuals is very rare in standardized and quality-assured laboratory settings [20]. Therefore, the result from our study corroborates that indeterminate results are apparently linked to the presence of rheumatic diseases. 

Considering that there is disagreement whether the use of an immunosuppressive agent is associated with an indeterminate IGRA result, a detailed analysis was undertaken to evaluate factors associated with this result. On evaluating the characteristics of the eleven patients with indeterminate results, we found that two of them (18.2%) were naïve to medications, while the remaining (81.8%) were on medications to treat underlying diseases. However, the logistic regression analysis revealed that glucocorticoid and immunosuppressive agent usage was not an independent predictive factor for indeterminate results. These results, at least in part, imply that the effect of drugs may not have a significant effect on indeterminate results among patients with rheumatic diseases.

A comparison of IGRA results between the QFT-GIT and QFT-PLUS groups showed that there was no significant difference observed regarding the results of both QFT tests. Subsequently, indeterminate results were shown to be inversely affected by lymphocyte counts and albumin levels. Interestingly, when adopting the optimal cut-off values for lymphocyte counts and albumin levels, it was found that the RR was significantly high for those with a lymphocyte count of ≤810/mm^3^ (RR 14.072) and an albumin level of ≤3.7 mg/dL (RR 12.041). Furthermore, for those with both lymphocyte counts and albumin levels below the cut-off limits, the RR was over 17 times higher. Accordingly, it could be suggested that attending physicians should be conscious of an indeterminate IGRA result being reported when lymphopenia or hypoalbuminemia is present.

Two possible interpretations could be considered regarding the association of lymphocyte counts and albumin levels with indeterminate IGRA results. First, indeterminate results of QFT-GIT and QFT-PLUS tests are usually affected by the decrease in IFN-γ production in the Mitogen tube or an increase in the Nil tube. Therefore, comparable results of QFT-GIT and QFT-PLUS tests may be due to the altered IFN-γ production in the Mitogen and Nil tubes in patients with rheumatic diseases. Given that IFN-γ is predominantly produced by T lymphocytes [21], diminished lymphocyte counts could be related to the decrease in IFN-γ production in Mitogen tubes, which leads to a higher probability of indeterminate results. This hypothesis is further supported by the fact that HIV-infected individuals with low CD4+ T cell counts have a high rate of indeterminate results [22,23]. Conversely, IFN-γ is a representative inflammatory cytokine, and albumin is an acute phase reactant that decreases in inflammation [24,25]. While increased IFN-γ production in the Nil tube represents a heightened systemic inflammatory process and causes indeterminate IGRA results, a reduction in the albumin level may indicate the severity of ongoing inflammation. Notably, a previous publication showed that a lower albumin level was related to indeterminate IGRA results [26]. Likewise, our results seem to be consistent with the literature that insists that both lymphocyte counts and albumin levels are important factors linked to indeterminate results in routine clinical practice [27]. Nonetheless, since pathophysiological evaluation leading to indeterminate results was not performed in this study, further investigations are essential in the future to better understand this phenomenon.

Several issues should be addressed as a limitation of this study. First, our study was retrospective in nature. Therefore, the collection of disease activity indices in rheumatic diseases (i.e., SLE Disease Activity Index for SLE, and Disease Activity Score 28 for RA) was not possible. In addition, indications for the QFT-GIT and QFT-PLUS tests may not be identical and might be subject to selection bias. Second, medications used to treat rheumatic diseases and disease activity could also influence the peripheral lymphocyte counts and affect the IGRA results. Third, due to the diversity of rheumatic diseases included in this study, we were not able to estimate the relative severity of the respective diseases precisely and assess the impact of the primary diagnosis on indeterminate results. Therefore, prospective studies with a large number of patients are necessary to better elucidate the factors affecting indeterminate IGRAs.

## 5. Conclusions

In conclusion, we found that the diagnostic performance between the QFT-GIT and QFT-PLUS tests was comparable in patients with rheumatic diseases. However, in clear contrast to healthy controls, patients with rheumatic diseases more frequently had indeterminate results. Additionally, a decreased lymphocyte count and albumin level should be recognized as factors contributing to indeterminate IGRA results, requiring careful consideration when providing rheumatological care.

## Figures and Tables

**Table 1:** Baseline characteristics of patients tested using QFT-GIT and QFT-PLUS.

	QFT-GIT Group (*n* = 177)	QFT-PLUS Group (*n* = 231)	*p*-Value
Demographics			
Age (years), mean ± SD	48.9 ± 15.4	47.8 ± 15.0	0.473
Sex, *n* (%)			0.036
Female	125 (70.6)	140 (60.6)	
Male	52 (29.4)	91 (39.4)	
Primary diagnosis, *n* (%)			0.052
Rheumatoid arthritis	81 (45.8)	79 (34.2)	
Ankylosing spondylitis	22 (12.4)	48 (20.8)	
Behcet disease	21 (11.9)	33 (14.3)	
Systemic lupus erythematosus	18 (10.2)	23 (10.0)	
Adult-onset Still disease	13 (7.3)	6 (2.6)	
Systemic necrotizing vasculitis	7 (4.0)	17 (7.4)	
Psoriatic arthritis	6 (3.4)	12 (5.2)	
Dermatomyositis/polymyositis	4 (2.3)	4 (1.7)	
Polymyalgia rheumatica	2 (1.1)	3 (1.3)	
Systemic sclerosis	2 (1.1)	2 (0.9)	
IgG4-related disease	1 (0.6)	4 (1.7)	
Laboratory data, mean ± SD			
White blood cell count (/mm^3^)	7661.6 ± 3921.2	7765.2 ± 3611.6	0.782
Neutrophil count (/mm^3^)	5222.2 ± 3347.4	5396.9 ± 4092.6	0.636
Lymphocyte count (/mm^3^)	1621.8 ± 976.2	1705.8 ± 920.0	0.374
Hemoglobin (g/dL)	12.4 ± 1.9	12.6 ± 2.0	0.221
Platelet count (×1000/mm^3^)	276.6 ± 120.1	286.5 ± 105.1	0.378
Blood urea nitrogen (mg/dL)	16.1 ± 9.2	16.9 ± 12.9	0.462
Creatinine (mg/dL)	0.9 ± 1.4	0.9 ± 0.9	0.557
Albumin (mg/dL)	3.9 ± 0.5	4.0 ± 0.6	0.751
Aspartate aminotransferase (IU/L)	22.4 ± 13.3	29.0 ± 80.5	0.219
Alanine aminotransferase (IU/L)	19.7 ± 16.4	24.6 ± 36.7	0.072
Erythrocyte sedimentation rate (mm/h)	49.1 ± 37.1	38.5 ± 32.4	0.002
C-reactive protein (mg/L)	23.7 ± 46.1	21.8 ± 42.9	0.681
IGRA results, *n* (%)			0.226
Indeterminate	4 (2.3)	7 (3.0)	
Positive	44 (24.9)	40 (17.3)	
Negative	129 (72.9)	184 (79.7)	

QFT-GIT: QuantiFERON-TB Gold In-Tube; QFT-PLUS: QuantiFERON-TB Gold PLUS; IgG4: immunoglobulin G4; SD: standard deviation; IGRA: interferon-gamma release assay.

**Table 2:** Difference in medication usage between the QFT-GIT and QFT-PLUS groups.

Medications, *n* (%)	QFT-GIT Group (*n* = 177)	QFT-PLUS Group (*n* = 231)	*p*-Value
Glucocorticoid			0.040
No	76 (42.9)	124 (53.7)	
Yes	101 (57.1)	107 (46.3)	
Methotrexate			0.241
No	104 (58.8)	150 (64.9)	
Yes	73 (41.2)	81 (35.1)	
Hydroxychloroquine			0.459
No	157 (88.7)	198 (85.7)	
Yes	20 (11.3)	33 (14.3)	
Sulfasalazine			0.452
No	131 (74.0)	162 (70.1)	
Yes	46 (26.0)	69 (29.9)	
Tacrolimus			0.028
No	162 (91.5)	224 (97.0)	
Yes	15 (8.5)	7 (3.0)	
Leflunomide			1.000
No	151 (85.3)	197 (85.3)	
Yes	26 (14.7)	34 (14.7)	
5-acetylsalicylic acid			0.798
No	166 (93.8)	214 (92.6)	
Yes	11 (6.2)	17 (7.4)	
Azathioprine			0.524
No	171 (96.6)	219 (94.8)	
Yes	6 (3.4)	12 (5.2)	
Mycophenolate mofetil			1.000
No	172 (97.2)	224 (97.0)	
Yes	5 (2.8)	7 (3.0)	
Cyclophosphamide			1.000
No	176 (99.4)	230 (99.6)	
Yes	1 (0.6)	1 (0.4)	
Cyclosporine			1.000
No	174 (98.3)	228 (98.7)	
Yes	3 (1.7)	3 (1.3)	
bDMARDs/tsDMARDs			0.353
No	162 (91.5)	218 (94.4)	
Yes	15 (8.5)	13 (5.6)	

QFT-GIT: QuantiFERON-TB Gold In-Tube; QFT-PLUS: QuantiFERON-TB Gold PLUS; bDMARDS: biologic disease-modifying antirheumatic drugs; tsDMARDs: targeted synthetic disease-modifying antirheumatic drugs.

**Table 3:** Indeterminate rates among patients with rheumatic diseases and healthy controls.

	QFT-GIT (RD) (*n* = 177)	QFT-PLUS (RD) (*n* = 231)	QFT-GIT (HC) (*n* = 2511)	QFT-PLUS (HC) (*n* = 1614)	*p*-Value
IGRA results, *n* (%)					<0.001
Indeterminate	4 (2.3)	7 (3.0)	5 (0.2)	4 (0.2)	
Positive/negative	173 (97.7)	224 (97.0)	2506 (99.8)	1610 (99.8)	

QFT-GIT: QuantiFERON-TB Gold In-Tube; QFT-PLUS: QuantiFERON-TB Gold PLUS; RD: rheumatic diseases; HC: healthy control.

**Table 4:** Description of patient characteristics with indeterminate IGRA results.

Patient	Age	Sex	Diagnosis	Lymphocyte Count (/mm^3^)	Albumin (mg/dL)	ESR (mm/h)	CRP (mg/L)	Current Medication Usage	IGRA Test	IFN-γ Level in Nil Tube	IFN-γ Level in Mitogen Tube
#1	49	F	DM	71	2.6	25	40.3	mPD 40 mg, TAC, RTX	QFT-GIT	0.04	0.06
#2	48	F	DM	640	3.5	95	47.9	mPD 12 mg, MMF, HCQ	QFT-PLUS	0.15	0.43
#3	68	M	DM	710	2.7	16	8.8	PL 10 mg	QFT-PLUS	0.02	0.12
#4	7	M	RA	1330	3.6	31	57.6	PL 20 mg	QFT-GIT	0.08	0.29
#5	54	F	RA	1460	3.7	120	36.7	MTX, HCQ	QFT-GIT	0.04	0.06
#6	69	F	RA	720	2.3	120	341.1	None	QFT-PLUS	0.04	0.44
#7	39	F	SLE	650	2.6	57	62.3	None	QFT-GIT	0.95	1.07
#8	36	F	SLE	30	2.9	24	35.6	PL 50 mg, HCQ	QFT-PLUS	≥10	≥10
#9	37	F	SLE	810	3.9	120	31.1	mPD 62.5 mg	QFT-PLUS	0.09	0.25
#10	31	M	SLE	1830	4.5	17	0.3	mPD 4 mg, MMF	QFT-PLUS	≥10	≥10
#11	47	F	SLE	260	2.2	116	114	PL 30 mg, MMF	QFT-PLUS	0.1	0.25

IGRA: interferon-gamma release assay; ESR: erythrocyte sedimentation rate; CRP: C-reactive protein; IFN-γ: interferon-γ; DM: dermatomyositis; mPD: methylprednisolone; TAC: tacrolimus; RTX: rituximab; QFT-GIT: QuantiFERON-TB Gold In-Tube; MMF: mycophenolate mofetil; HCQ: hydroxychloroquine; QFT-PLUS: QuantiFERON-TB Gold PLUS; PL: prednisolone; RA: rheumatoid arthritis; MTX: methotrexate; SLE: systemic lupus erythematosus.

**Table 5:** Logistic regression analysis for factors associated with indeterminate results.

	Univariate Analysis			Multivariate Analysis ^‡^		
Variables	HR	95% CI	*p*-Value	HR	95% CI	*p*-Value
Age	0.982	0.944, 1.021	0.353			
Female sex	0.688	0.180, 2.636	0.586			
White blood cell count	1.000	1.000, 1.000	0.461			
Neutrophil count	1.000	1.000, 1.000	0.109			
Lymphocyte count	0.998	0.996, 0.999	<0.001	0.998	0.997, 1.000	0.012
Hemoglobin	0.592	0.442, 0.793	<0.001			
Platelet count	0.992	0.985, 0.998	0.013			
Blood urea nitrogen	1.013	0.976, 1.053	0.492			
Creatinine	0.936	0.483, 1.812	0.844			
Albumin	0.220	0.105, 0.463	<0.001	0.366	0.150, 0.890	0.027
Aspartate aminotransferase	1.013	0.998, 1.027	0.081			
Alanine aminotransferase	1.011	1.003, 1.019	0.009			
Erythrocyte sedimentation rate	1.018	1.002, 1.034	0.026			
C-reactive protein	1.010	1.004, 1.017	0.002			
Glucocorticoid usage	2.627	0.687, 10.046	0.158			
Immunosuppressive agent usage	0.372	0.111, 1.247	0.109			

^‡^ Including statistically significant variables in univariate analysis only. HR: hazard ratio; CI: confidence interval.

## Data Availability

The datasets used and/or analyzed during the current study are available from the corresponding author on reasonable request.
